# Dual Dynamic Scheduling for Hierarchical QoS in Uplink-NOMA: A Reinforcement Learning Approach

**DOI:** 10.3390/s21134404

**Published:** 2021-06-27

**Authors:** Xiangjun Li, Qimei Cui, Jinli Zhai, Xueqing Huang

**Affiliations:** 1 National Engineering Laboratory for Mobile Network Technologies, Beijing University of Posts and Telecommunications, Beijing 100876, China; xiangjunli@bupt.edu.cn (X.L.); chenest@bupt.edu.cn (J.Z.); 2 New York Institute of Technology, Old Westbury, NY 11568, USA; xhuang25@nyit.edu

**Keywords:** deep deterministic policy gradient (DDPG), hierarchical QoS, nonorthogonal multiple access (NOMA), power allocation, reinforcement learning (RL)

## Abstract

The demand for bandwidth-intensive and delay-sensitive services is surging daily with the development of 5G technology, resulting in fierce competition for scarce radio resources. Power domain Nonorthogonal Multiple Access (NOMA) technologies can dramatically improve system capacity and spectrum efficiency. Unlike existing NOMA scheduling that mainly focuses on fairness, this paper proposes a power control solution for uplink hybrid OMA and PD-NOMA in dual dynamic environments: dynamic and imperfect channel information together with the random user-specific hierarchical quality of service (QoS). This paper models the power control problem as a nonconvex stochastic, which aims to maximize system energy efficiency while guaranteeing hierarchical user QoS requirements. Then, the problem is formulated as a partially observable Markov decision process (POMDP). Owing to the difficulty of modeling time-varying scenes, the urgency of fast convergency, the adaptability in a dynamic environment, and the continuity of the variables, a Deep Reinforcement Learning (DRL)-based method is proposed. This paper also transforms the hierarchical QoS constraint under the NOMA serial interference cancellation (SIC) scene to fit DRL. The simulation results verify the effectiveness and robustness of the proposed algorithm under a dual uncertain environment. As compared with the baseline Particle Swarm Optimization algorithm (PSO), the proposed DRL-based method has demonstrated satisfying performance.

## 1. Introduction

Power domain Nonorthogonal Multiple Access (NOMA) has introduced power multiplexing into four standard dimensions of the wireless communications systems: time, frequency, code, and space, thereby greatly improving spectrum efficiency and capacity [1]. As a trend of 6G network development, the performance of NOMA depends on user pairing, power allocation, and detection-decoding, which are closely related to NOMA performance [2]. For serious interference problems caused by the reuse of frequency resources, advanced physical layer and multiuser detection techniques such as serial interference cancellation (SIC) is applied at the receiver. In addition, hybrid OMA and NOMA can also reduce interference between users. User groups follow OMA channel allocation, while internal users perform NOMA.

At present, there are three methods commonly used to solve the NOMA power allocation problem: Convex Optimization, Game Theory, and Reinforcement Learning.

The Convex Optimization method requires full knowledge of the environment and problem convexity. The Game Theory method focuses on game behavior between users but neglects the environment. However, due to the dual uncertainty in wireless network environments: channel as well as QoS requirements change with users and time; these conventional model-based approaches that require the complete knowledge of systems and high computational complexity can be inefficient or even infeasible in practice. With the model-free method of Reinforcement Learning (RL) through continuous interaction with the environment, the strategy can also be improved, eliminating the need for the modeling process. Many researchers try to apply RL technology to network contexts, including dynamic IoT networks routing [3], MEC offloading [4], security, and so on [5].

For the above three methods to solve the power distribution problem under NOMA, as well as the deficiencies of the existing RL-based methods, please refer to Section 2.

Based on this, this research proposes an uplink power allocation algorithm under hybrid NOMA. It considers the environment under dual uncertainty, which means imperfect time-varying channel information and random users’ hierarchical QoS requirements. The problem is a nonconvex, stochastic, and NP-hard problem, which is then formulated as a partially observable Markov decision process (POMDP). Therefore, this research uses the DDPG algorithm to schedule uplink power under a dual uncertain environment without manual derivation of the problem and environment modeling. Moreover, this research transforms the hierarchical QoS to minimum power constraint under the SIC scene. Simulation results show that the proposed approach can achieve satisfying hierarchical QoS with less energy consumption, faster convergence speed, as well as robustness under a dual uncertain environment. Moreover, the proposed algorithm achieves comparable performance close to the global optimum with low computational time complexity. Further, the DDPG-based uplink NOMA is far better than the baseline algorithm. The main contributions of this paper are as follows:

- This paper uses hierarchical QoS to characterize the different service requirements of users, and expresses them with minimum rate requirements;
- This paper transforms the hierarchical QoS to minimum power constraint under the SIC scene and adds a penalty term to the real-time return in the Reinforcement Learning to represent the QoS requirement;
- Considering the dual dynamics of the channel and user requirements, nonconvex optimization problem, this paper proposes a DDPG-based method;
- Simulation results show that compared with the global search algorithm PSO, the DDPG method is more adaptable to dynamic environments and has a faster convergence speed.

This paper is organized as follows: Section 2 gives a brief overview of the state of the art. Section 3 gives the system model and constructs the power allocation problem. In Section 4, this paper briefly introduces the DDPG algorithm and then explains the details of the power control algorithm. In Section 5, the simulation results are shown and analyzed. Section 6 summarizes the work of this paper.

## 2. Related Work

In [6], the authors applied successive convex approximation to solve the power allocation problem. Pang et al. allocated downlink power to maximize energy efficiency. The solution to it includes outer iteration and inner iteration, fractional programming, and successive convex optimization [7]. Chen et al. considered the same problem under short packet communication. It is modeled as a nonconvex mixed integer nonlinear problem (MINLP), which is solved by a block coordinate descent algorithm [8]. The quality of service (QoS) requirement is further considered in [9], which renders the power allocation problem as stochastic and quasi-concave. The constrained problem is iteratively solved by the bisection research algorithm. In [10], the power allocation problem was transformed into the dual Lagrangian problem using the subgradient algorithm in MIMO and NOMA downlink scenarios. JIAO et al. proposed a fairness-improved and QoS-guaranteed resource allocation for the S-IoT NOMA downlink network, exploiting the Lyapunov framework to break down the nonconvex joint optimization problem into a sequence of individual subproblems. Further, they used the particle swarm optimization (PSO) algorithm to solve the proposed subproblems [11].

Li et al. proposed an energy-efficient resource allocation scheme with hybrid TDMA–NOMA for cellular-enabled M2M networks. They formulate the problem as a noncooperative game and transform the nonconvex optimization problem into the convex form by using nonlinear fractional programming and solve the transformed problem by Dinkelbach’s method and Lagrangian duality theory [12]. Adjif et al. adopted a multiarmed bandit-based method (MAB) in the uplink scenario with the goal of optimizing system throughput [13]. Zheng et al. considered user selfishness, and modeled the NOMA uplink power allocation problem as a Nash bargaining game, which is solved by KKT Condition [14]. Aldebes et al. aimed at maximizing the sum rate in the downlink NOMA cellular system. Glicksberg game-based algorithm is used to allocate the power between different numbers of users [15]. Omslandseter et al. considered the problem of power allocation as a variation of the Knapsack Problem, and solved it through a greedy solution [16].

RL is built between the base station (BS) and the user based on Contract Theory in heterogeneous uplink NOMA and imperfect CSI [17]. The actor–critic algorithm is used to control downlink NOMA and maximizes the sum of the user’s rate [18]. Yet, the actor–critic network has the problem of convergence. Zhang et al. used the deep reinforcement learning (DRL) algorithm DDPG to maximize the sum of user energy efficiency [19]. In [20], hardware sensitivity and imperfect successive interference cancellation (SIC) are considered. Additionally, a multiagent structure and a convolutional neural network are adopted to reduce the complexity of the power allocation in NOMA. The authors of [21] proposed asynchronous reinforcement learning-based schemes to solve joint relay selection and power allocation, which is a complicated high-dimensional optimization problem. In the above, the scheduling variable in some references is discrete, resulting in quantization errors, and they only discuss single QoS for all users while ignoring the multiple QoS requirements brought by differentiated services, i.e., hierarchical QoS, such as delay-jitter-sensitive services and instant messaging services.

## 3. System Model and Problem Formulation

**Figure 1:**
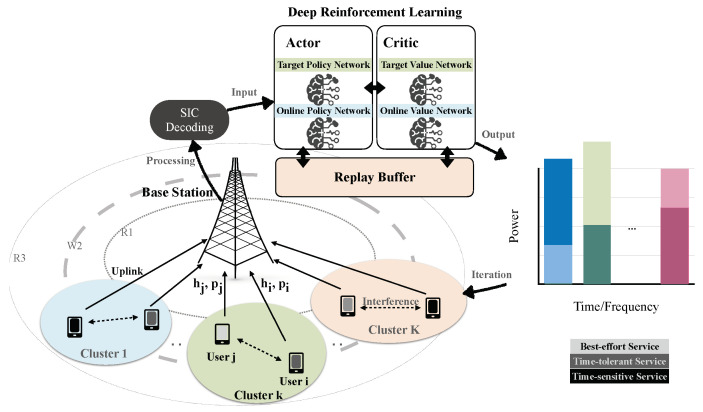
Multiuser with hierarchical QoS in Uplink NOMA network and Reinforcement Learning for power allocation.

The issue of hybrid OMA and NOMA uplink power control is studied for the system shown in Figure 1. A single-antenna base station (BS) is equipped with an SIC module. M users are uniformly distributed in the coverage area of the BS, i.e., between two circles with radius $R_{1}$ and $R_{3}$, where the inner radius $R_{1}$ is used to simulate the minimum propagation path loss, and the outer radius $R_{3}$ represents the cell size [22]. In between, a ring with width $w_{2}$ splits near and far users. The scattered users are first clustered into K groups by distance. Then, orthogonal time-frequency resources are allocated among the groups, and users within the same group reuse the same time-frequency resource block (no mutual interference among groups). The reinforcement learning agent at the BS side adjusts the user’s uplink power by considering the dynamic imperfect uplink channel information. The goal of the RL agent is to maximize the system energy efficiency while meeting the use-specific QoS requirement in terms of throughput. For the convergence of the RL agent, the multiple minimum rate thresholds are used to characterize the user’s QoS requirements and can be transformed into a minimum power constraint under the SIC scene for convergence of RL.

### 3.1. Network Framework and Objective Function

Suppose *M* users are clustered into *K* user groups, and each group supports up to $N_{pair}=2$ users to reuse the same time-frequency resource block $R_{b}$ [23]. The total bandwidth of the BS is B, namely, the bandwidth of each group is B/K. For simplicity, this research assumes $M=N_{pair}*K$. The Shannon Capacity formula is shown in (1), where $\gamma_{i,t}$ represents the Signal-to-Interference-plus-Noise Ratio (SINR) of the *i*-th user at time *t*, and $r_{i,t}$ is the corresponding Shannon Capacity of the *i*-th user. Please note that the dual dynamics mentioned in this article refer to the channel gain *h*, and user requirements $r^{min}$ will change over time *t*.

(1) $$r_{i,t}=\frac{B}{K}\log_{2}(1+\gamma_{i,t}),\;i\in M.$$

Expansion of $\gamma_{i,t}$ is in (2), where $p_{i,t},d_{i},g_{i,t}$ are the uplink power of the *i*-th user, the distance between the user and the BS, and the small-scale channel fading. *n* presents additive white Gaussian noise, and α is the path loss exponent. The interference from the other user sharing time-frequency resources denotes as $I_{i,t}$.

(2) $$\gamma_{i,t}=\frac{p_{i,t}d_{i}^{-\alpha }g_{i,t}}{n+I_{i,t}},\;i\in M.$$

Assume the *i*-th user is paired with the *j*-th user (the details of the user pairing algorithm is given in Section 3), the interference experience by user *i* can be simplified to (3) according to the power descending demodulation sequence of SIC.

(3) $$I_{i,t}=\{\begin{array}{cc} 0 & \mathrm{if}\;p_{i,t}\leq p_{j,t},\\ p_{j,t}d_{j}^{-\alpha }g_{j,t}\; & \mathrm{if}\;p_{i,t}>p_{j,t}. \end{array}$$

System Energy efficient (*EE*) at time *t* is defined in (4), where U(·) is the step function, $p_{0}$ denotes base station circuit power consumption, and the *j*-th user pairs with the *i*-th user.

(4) $$\begin{array}{cc} EE & =\sum_{i=1}^{M}\frac{r_{i,t}}{p_{i,t}+p_{0}}\\ \; & =\sum_{i=1}^{M}\frac{B}{K(p_{i,t}+p_{0})}\log_{2}(1+\frac{p_{i,t}d_{i}^{-\alpha }g_{i,t}}{n+U(p_{i,t}-p_{j,t})p_{j,t}d_{j}^{-\alpha }g_{j,t}}). \end{array}$$

### 3.2. Optimization Problem and QoS Constraint Transformation

Considering the user-specific throughput requirement and the uplink power constraints of users, the mathematical optimization model to maximize energy efficiency is given as follows:

(5)maxpiEE(6)s.t.0≤pi,t≤Pmax,∀i∈M(7)ri,t≥ri,tmin,∀i∈M.

Once the users are paired, the optimization variable is user’s uplink power $p_{i,t},\forall i\in M$. This is a nonconvex continuous variable random optimization problem. $P_{max}$ represents the user-common maximum uplink power of the user, and the value is the same for all users. In this paper, the threshold rate $r_{i,t}^{min}$, varying with user and time, is used to characterize the i-th user QoS requirements. In addition, the small-scale fading $g_{i,t}$ remains unchanged within a period and changes with the user and the time slot.

Among them, (7) can be further simplified. Let $r_{i,t}^{min}$ and $r_{j,t}^{min}$ denote the minimum rate requirements of the two users i,j in the same group, respectively. The channel gain of the two users is $h_{i,t}=d_{i}^{-\alpha }g_{i,t},h_{j,t}=d_{j}^{-\alpha }g_{j,t}$, assuming $p_{i,t}\leq p_{j,t}$, B/K=1. According to (3), $I_{i,t}=0,I_{j,t}=p_{i,t}h_{i,t}$. Substituting into (1) and (2), we obtain

(8) $$r_{i,t}=\log_{2}(1+\frac{p_{i,t}h_{i,t}}{n})$$

(9) $$r_{j,t}=\log_{2}(1+\frac{p_{j,t}h_{j,t}}{n+p_{i,t}h_{i,t}}).$$

Further simplification can be obtained by monotonicity.

(10) $$\begin{array}{cc} p_{i,t}^{min} & =\frac{n(2^{r_{i,t}^{min}}-1)}{h_{i,t}},\;\mathrm{if}\;p_{i,t}\geq p_{i,t}^{min} \end{array}$$

(11) $$\begin{array}{cc} p_{j,t}^{min} & =\frac{2^{r_{j,t}^{min}}-1}{h_{i,t}}\cdot (n+p_{i,t}h_{i,t}) \end{array}$$

Substitute (10) into (11).

(12)pj,tmin≥2rj,tmin−1hj,t·(n+n(2ri,tmin−1))(13)=2rj,tmin−1hj,t·n·2ri,tmin.

From (10) and (13), it can be seen that the minimum rate constraint can be transformed into a minimum power limit. Then, the optimization problem after the constraint transformation is given below.

(14)maxpi,t,pj,t,pi,t≤pj,tEE(15)s.t.n2ri,tmin−1hi,t≤Pi,t≤Pmax(16)2rj,tmin−1hj,t·n·2ri,tmin≤Pj,t≤Pmax.

This section establishes an optimization model, which will be solved below.

## 4. Algorithm

### 4.1. Pairing of Near and Far Users

The optimal pairing algorithm is used to traverse all the combinations, select the one with the largest EE, distribute the power again, re-pair, and iterate until convergence. In this paper, the control variable method is used to study the convergence performance and speed of the DRL-based power allocation strategy given a specific pair under a dual uncertainty environment. Therefore, the user pairing algorithm is simplified to the basic far and near user pairing. First, sort the users according to distance, $d_{1}\leq \cdots \leq d_{i}\cdots \leq d_{m}$—that is, the user numbered 1 is the closest to the BS. Then, match users at equal intervals.

### 4.2. Deep Deterministic Policy Gradient (DDPG)

DDPG was developed to deal with continuous space problems enabled by neural network approximation capability [24]. Since both the channel state and the power value are continuous variables, this research chooses the DDPG algorithm. DDPG algorithm based on Actor–Critic architecture has two networks to ensure stability, namely, Online Network and Target Network. It also adds noise to the Actor Network output to tackle the exploration problem. Among them, Online Actor and Critic network parameters are denoted as $\theta^{\mu }$ and $\theta^{Q}$. The parameters of these two target networks are represented by $\theta^{\mu^{t}}$ and $\theta^{Q^{t}}$. The target networks use the Poylak average with a parameter of τ to soft update in (17).

(17) $$\begin{array}{cc} \; & \theta^{Q^{t}}\leftarrow \tau \theta^{Q}+\left(1-\tau \right)\theta^{Q^{t}}\\ \; & \theta^{\mu^{t}}\leftarrow \tau \theta^{\mu }+\left(1-\tau \right)\theta^{\mu^{t}}. \end{array}$$

Actor Network determines the deterministic mapping from state $s_{t}$ to action $a_{t}$, i.e., $S\Rightarrow A,a_{t}=\mu \left(s_{t}|\theta^{\mu }\right)$, instead of outputting probability distributions in discrete action spaces. The objective function of Actor Network is in (18).

(18) $$J(\mu_{\theta })=\int_{s}\rho \left(s\right)V^{\mu }\left(s\right)ds=\int_{s}\rho \left(s\right)Q^{\mu }(s,\mu_{\theta }\left(s\right))ds.$$

In between, ρ(s) represents the state probability distribution; further, on behalf of the state-value, $V^{\mu }\left(s\right)$ is equal with action-value $Q^{\mu }(s,\mu_{\theta }\left(s\right))$ because of deterministic policy. In the meantime, the Critic Network implements the mapping from state-action pair $\left(s_{t},a_{t}\right)$ to value $Q(s_{t},a_{t}|\theta^{Q})$, S,A⇒Q. Value represents the prediction of future environment total return using the Bellman equation, as given below. *R* is the experience pool for the i.i.d example, and γ is the discount factor.

(19) $$Q^{\mu }(s_{t},a_{t})=E_{r_{t},s_{t+1}∼R}[r(s_{t},a_{t})+\gamma Q^{\mu }(s_{t+1},\mu (s_{t+1}))].$$

### 4.3. Learning Agent Design: State, Action and Reward

According to the interaction between the agent and the environment, this paper designs the incompletely observed environmental state $s=\{h_{1},...,h_{K},r_{1}^{\;min},...,r_{K}^{\min}\}$, environmental action $a=\{p_{1},p_{2},\ldots ,p_{K}\}$, and instant return function $r=EE-\sum_{i=1}^{M}\beta \cdot {\left(max\left(P_{i}-P_{i}^{min},0\right)\right)}^{2}$. The second term of the return represents the power constraint. Due to the sigmoid function of the action network, the output power will not exceed the maximum power constraint, but the minimum power constraint derived from QoS is not guaranteed. The literature [17,18,19] adopt a stepwise return—that is, there is a return when the constraint conditions are met, and the return is 0 or a constant if the constraint is not met. However, through simulation verification, this setting is difficult to converge, so the penalty function setting is used to make the reward function more continuous and easy to converge.

**Figure 2:**
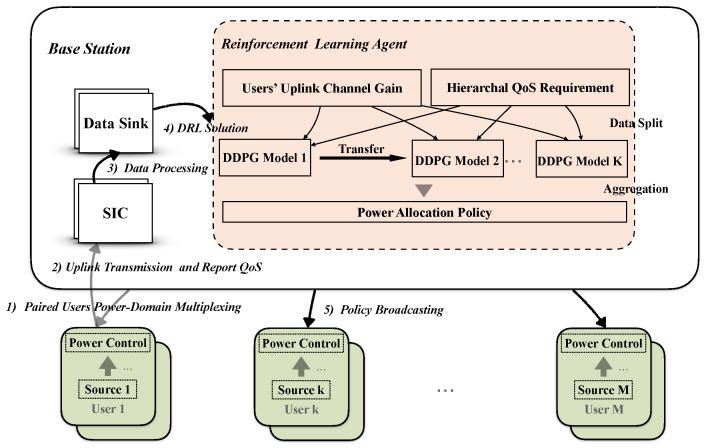
Flow chart of the Deep Deterministic Policy Gradient.

Figure 2 introduces the process of the uplink NOMA power allocation procedure. Based on random channel state and unequal user demand vector, this research aims to obtain the best possible policy of user uplink transmit power. The detailed descriptions of each step are as follows:

1. After the users are paired, users in the same group can adopt power multiplexing on the same time-frequency resources;
2. Users periodically report their QoS requirements while transmitting data in the uplink return under the previous action (report reward during training);
3. After the continuous interference cancellation on the base station side, according to the previous derivation, the QoS is converted into the minimum power requirement;
4. The data is classified into user groups and then input into the DDPG module. Each group of users uses a DDPG module, which not only guarantees user scalability but also uses the idea of parallel computing to speed up decision-making. It should be noted that only one DDPG module is involved in model training. After convergence, the model Actor Network can be directly copied to process multiple sets of user scenarios;
5. The last step is to broadcast the strategy to each user (collect the current action return and observe the state after the transfer during training).

## 5. Simulation

### 5.1. Simulation Parameters

To verify the effectiveness of the proposed scheme, the transmission and convergence performance of the proposed scheme is simulated. Table 1 summarizes important parameters in the simulation setup. The parameters in Table 1 are divided into two parts, one part is communication-related parameters [12,22,23] and the other part is reinforcement-learning-related parameters [24].

As the 4G subframe length is 20 ms [25] and reinforcement learning needs to obtain enough samples in the current unchanged environment, the interaction cycle between the RL agent and the environment is one-twentieth of Tf, which is 1 ms. The user’s minimum rate requirement is customized based on the $\log_{2}3\approx 1.585$ bit/s/Hz when the user’s signal-to-noise ratio is 3 dB, so the minimum rate is set by itself to be an integer multiple of 1.5.

In this paper, the minimum power requirement is derived from the minimum rate, and then reflected in the penalty term in the calculation of the instant rewards of reinforcement learning. Therefore, in the face of the actual minimum speed required by different business scenarios, the solution in this paper is also effective.

To simplify the simulation and result analysis, this research makes the following settings. (1) The channel gain consists of large-scale fading and small-scale fading, where the latter obeys the exponential distribution g∼E(1) and changes every $T_{f}$. (2) The user needs $r_{min}$ change every $T_{f}$, and chooses one level with the same probability. There are three types of $r_{min}$, which are represented by 1, 2, and 3. As the value increases, the rate constraint becomes tighter and tighter. (3) The interaction cycle between the agent and the environment is $T_{i}=1\;\mathrm{ms}$. After every $T_{f}$, the agent will resample the channel and user demand. The four networks in DDPG contain two hidden layers with 400 and 300 neurons, respectively, and use the ReLU activation function. Besides adding a sigmoid function to the output layer of the Actor Network (and its target network), the range of environmental actions is limited through reward penalty. The Power allocation algorithm is given in Algorithm 1.
**Algorithm 1:** Uplink Power Allocation Based on DDPG in NOMA
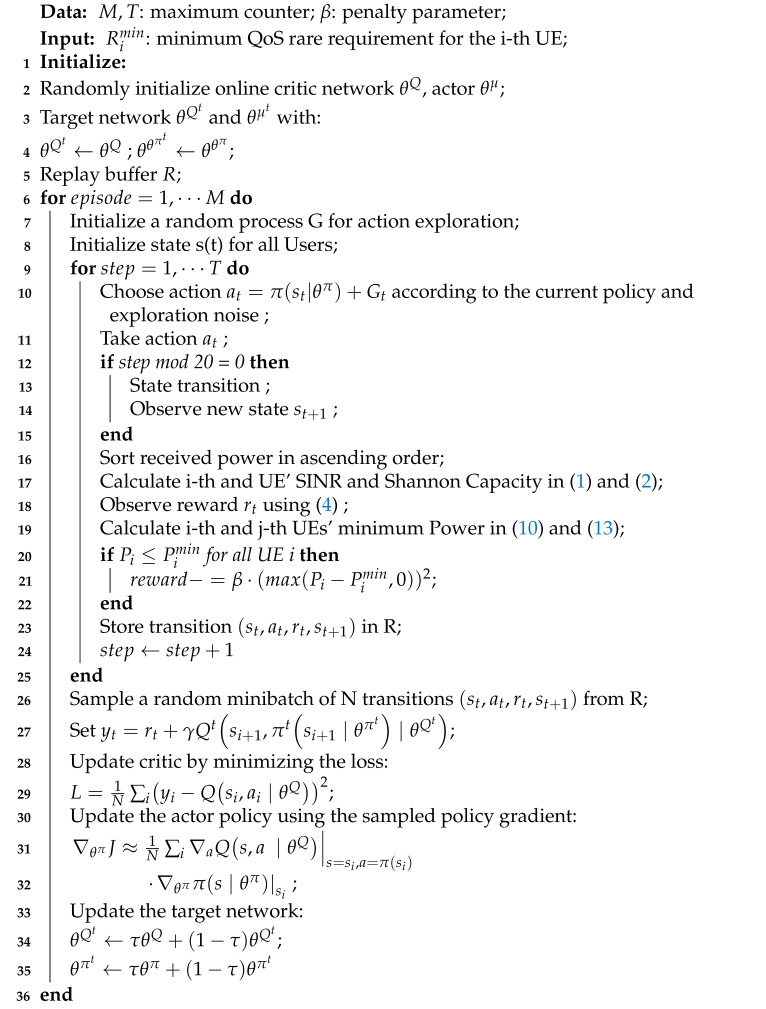


The baseline power allocation scheme adopted for performance comparisons includes the following: (1) the PSO algorithm, to compare the gap between the proposed algorithm and the optimal global solution. When the PSO’s running time tends to infinity, due to its randomness, it is bound to be the global optimal. It should be noted that the optimal solution solved by PSO is obtained in a static environment, i.e., both the channel and user’s QoS remain unchanged. To reflect the superiority of the artificial intelligence control algorithm, this research considers the (2) greedy strategy, where each user transmits in the maximum power to maximize self-interest; and (3) random strategy, where users select a random power value to transmit.

### 5.2. Simulation Result

In the following analysis, we evaluated the scheme from the perspective of instantaneous reward. As judging the system’s performance only depends on the current return, i.e., system energy efficiency and subsequent performance stability. this paper first simulates a pair of users with various user requirement combinations to verify its effectiveness. Then, this paper increases the number of users to test the proposed scheme.

**Figure 3:**
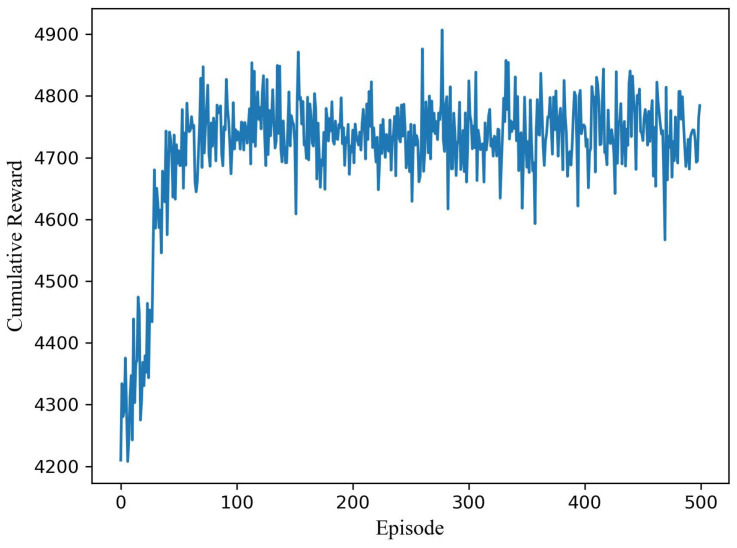
Optimization process for a pair of users under dynamic channel and hierarchical requirements.

Figure 3 shows the optimization process for a pair of users (user number M = 2, group number K = 1). The simulation results show that DDPG can converge quickly under a dual dynamic environment. Dynamic channel and user demand can be treated as disturbances under the static optimal strategy. After about 90 episodes, the algorithm can reach stability under different QoS combinations. There are 500 rounds in total, and each episode contains 2000 steps. Each step corresponds to a real-time of 1 ms so that the algorithm will converge within 180 s. The convergence time will be shortened if we enhance the parameter update frequency—that is, each episode contains fewer steps resulting in the surge of data utilization (Parameters are updated once every episode.).

**Figure 4:**
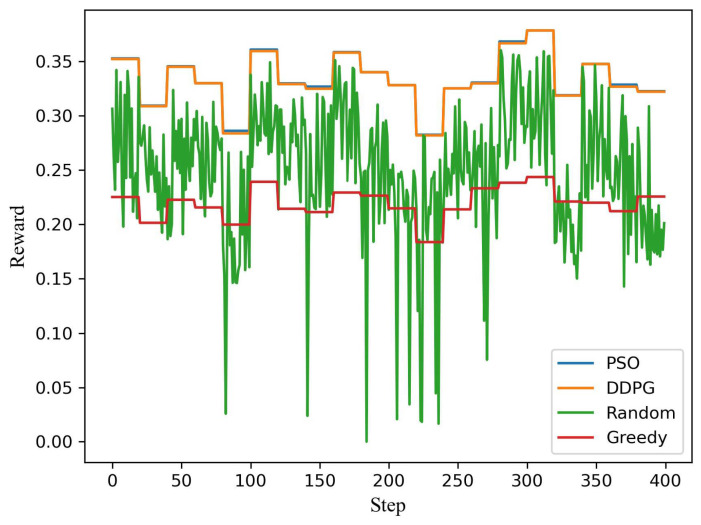
The system reward distance for each step for a pair of users.

Figure 4 shows the testing of the model under the dynamic channel and user demand. The test runs 400 steps, and the vertical axis is the reward for each step. There are four schemes (PSO, DDP, Random, and Greedy). The population size of the PSO algorithm is 10,000, and the number of iterations is 100. DDPG and PSO algorithms have similar and far better performance than the other two schemes (Random and Greedy). A penalty value in reward is nonzero if the user’s demand is not satisfied. However, the reward is always greater than or equal to zero. Therefore, it can be seen that a random scheme often reaches zero. From the above simulation, it can be seen that the DDPG algorithm can efficiently solve the power allocation of 2 users with time-varying demands assembly. The following scenario is simulated with the random demands of 10 users to analyze the user scalability of the algorithm.

**Figure 5:**
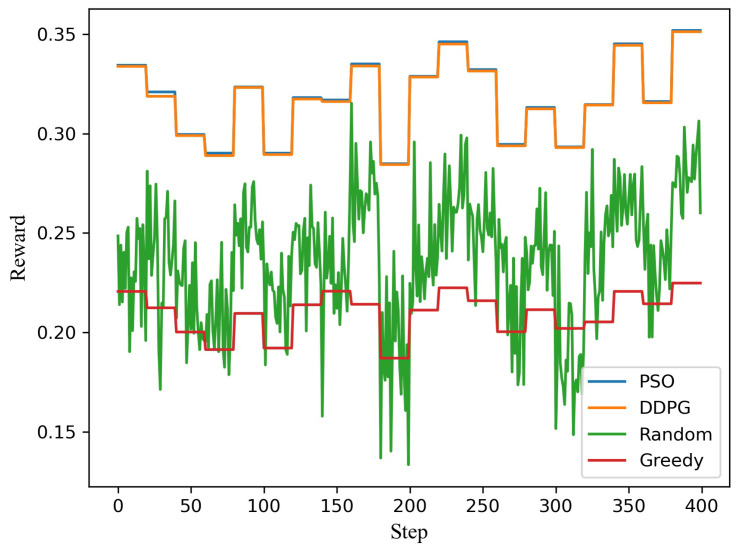
The system reward distance for each step for 5 pairs of users, comprising 10 users in total.

Taking into account the characteristics of Hybrid NOMA, when the number of user groups *K* is greater than 1, because the groups are independent of each other, the multiuser group problem can be split into independent subproblems and solved in parallel. According to the simulation above, the multiuser model based on DDPG likewise stabilizes with about 90 episodes—the same as the two-user model—at the cost of increasing the DDPG neural networks. Figure 5 shows the model under the scenario of 10 users’ random demands under a dynamic channel. As seen from the figure, the simulation phenomenon is very similar to that of two users, dealing with five subproblems simultaneously (dividing ten users into five pairs). When the number of users increases, the gap between the two algorithms is slightly larger than when the number of users is two because the gap in each subproblem is superimposed.

When the PSO’s running time tends to infinity, due to its randomness, it is bound to be the global optimal. However, slow convergence is its main flaw, especially in a dynamic and uncertain environment. According to the simulation, the time of the PSO algorithm is at least ten times that of the DDPG algorithm and, as the number of users increases, the time increases to more than 100 times. We assume that on a long-term scale, the environment and user behavior are regular. Unlike the neural network parameters in DRL that can retain a certain degree of memory, when the environment changes, the PSO needs to be recalculated. Equivalent to each change, the previous results will be discarded, then the PSO will be overturned and restarted. From the above simulation, it can be seen that the power allocation based on the DDPG algorithm is capable for different user needs and different user numbers with similar performance to PSO. Nevertheless, the time and space complexity are far less than the PSO algorithm, especially in the single-step computing resource occupation.

## 6. Conclusions

This paper studies the uplink power allocation scheme to optimize the energy efficiency for the NOMA system. To characterize diverse service requirements, this paper also introduces the hierarchical QoS constraints and transforms them into the corresponding transmission power thresholds. The proposed power allocation algorithm considers both the time-varying channel and the random hierarchical QoS requirements. With the highly dynamic and partially observed environment and the unbearable time complexity of the traditional optimization algorithm, the proposed DDPG-algorithm-based power allocation scheme can efficiently solve the energy efficiency optimization problem. Verified by the simulation results, the DDPG-based method can adapt to the dual uncertain environment within a low time complexity and obtain a result second only to the global optimal solution.

## Figures and Tables

**Table 1:** Important parameters in the simulation setup.

Parameter	Value
Discounted factor	γ=0.1
Large-scale fading exponent	α=−3
Small-scale fading exponent	1
Channel bandwidth	B=10 MHz
The number of users	M=10
The user power	P∈[0,40] mW
Learning rate	5 × 10${}^{-4}$
Target smoothing coefficient	τ=0.005
Replay buffer capacity	R = 1 × 10${}^{6}$
Minibatch size	512
Interaction cycle	$T_{i}=1$ ms
State sample interval	$T_{f}=20$ ms
Noise factor of AWGN channel	−174 dBm/Hz
Minimum rate threshold	$r_{min}=1.5*\left\{1,2,3\right\}$ bit/s/Hz
Base station circuit power	$p_{0}=40$ mW
Penalty parameter	β=9 × 10${}^{-3}$
Two rings range	$\left\{\left(100,200\right),\left(400,500\right)\right\},R_{1}=100,R_{3}=500,W_{2}=200$

## Data Availability

Not applicable.
